# Antifibrotic effect by activation of peroxisome proliferator-activated receptor–γ in corneal fibroblasts

**Published:** 2009-11-10

**Authors:** Hongwei Pan, Jiansu Chen, Jintang Xu, Miaojiao Chen, Rong Ma

**Affiliations:** 1 Department of Ophthalmology, Medical College, Jinan University, Guangzhou, China; 2 Key Laboratory for Regenerative Medicine of Ministry of Education, Jinan University, Guangzhou, China; 3 Medical college, Jinan University, Guangzhou, China

## Abstract

**Purpose:**

The transformation of quiescent keratocytes to active phenotypes and the ensuing fibrotic response play important roles in corneal scar formation. This study aims to observe the antifibrotic effect of peroxisome proliferator-activated receptor-γ (PPARγ) agonist on corneal fibroblasts cultured in vitro, and to explore the potential application of peroxisome proliferator-activated receptor agonist to the prevention of corneal opacity following wound repair.

**Methods:**

Rabbit corneal keratocytes were cultured in a medium containing 10% serum to induce their transformation to fibroblasts and myofibroblasts, which are similar to those that repair corneas. After incubation with the PPARγ agonist pioglitazone at different concentrations, the effect of pioglitazone on the migration, contractility, and viability of corneal fibroblasts was examined. The secretion of matrix metalloproteinase-2 and matrix metalloproteinase-9 was determined by gelatin zymography, and the synthesis of collagen I and fibronectin was investigated by western blotting.

**Results:**

Treatment with pioglitazone at concentrations ranging from 1 to 10 μm significantly decreased corneal fibroblast migration, as determined by scrape-wound assay, inhibited corneal fibroblast-induced collagen lattice contraction, and reduced MMP-2 and MMP-9 secretion into the supernatant of cell cultures in a dose-dependent manner. The expression of fibronectin was significantly decreased, while the expression of collagen I was only decreased when treated with 10 μm pioglitazone. Cell viability was not evidently changed compared to the control.

**Conclusion:**

This in vitro study demonstrated the anti-fibrotic effect of pioglitazone, suggesting that activation of PPARγ may be a new approach for the treatment of corneal opacity and scar formation in the corneal wound healing process.

## Introduction

The cornea is a highly specialized transparent tissue located at the anterior-most surface of the eye. As one component of the refractive media, the transparency of the cornea is very important for the maintenance of normal vision. However, once the cornea is in an injured condition resulting from, for example, infection, trauma, and surgery, it will undergo a repair process involving an inflammation reaction and a fibrotic response, which usually results in corneal opacity and scar formation. According to an epidemiological survey carried out in China, corneal scars have become the primary reason for keratoplasty. Moreover, the occurrence of haze following refractive surgery is believed to be related to the myofibroblasts that appear during the wound healing process [1]. Therefore, research on how to reduce the corneal scar formation by regulating the fibrotic response to injury will be of great clinical value for the improvement of the visual outcomes of patients suffering from corneal injury or receiving corneal surgery.

The corneal wound healing process involves a very complex and sometimes unpredictable biological response. The normally quiescent keratocytes are activated and transformed into fibroblasts and myofibroblasts under the stimulation of many inflammatory/fibrogenic growth factors or cytokines such as TGFβ, CTGF, and so on [2-4]. This in turn leads to increased extracellular matrix production, the altered arrangement and contraction of collagen fibril [5,6], and tissue remodeling of corneal stroma due to activation of various collagenases and other proteases [7,8]. Thus, keratocytes and their active phenotypes, including fibroblasts and myofibroblasts, play central roles in corneal fibrotic response and scar formation.

In recent years, many studies have demonstrated that peroxisome proliferator-activated receptor-γ (PPAR-γ) is involved in the anti-fibrotic effect in many tissues, such as the kidney [9], liver [10], pancreas [11,12], lung [13], and heart [14]. It is thought to be a promising target for the treatment of fibrotic diseases. The aim of this work was to investigate the effect of the PPARγ agonist, pioglitazone, on the function of corneal fibroblasts cultured in vitro. We demonstrated that pioglitazone inhibited cell migration, contractility, matrix metalloproteinase (MMP) secretion, and extracellular matrix production, probably in a non-cytotoxic way, suggesting that pioglitazone may exert a direct antifibrotic effect and have a potential use in the treatment of corneal scar formation.

## Methods

### Materials

Dulbecco’s Modified Eagle’s Medium, fetal bovine serum (FBS), and trypsin-EDTA were obtained from Invitrogen-Gibco (Carlsbad, CA); 6-well, 24-well, and 96-well culture plates, as well as 25 cm^2^ cell culture flasks were from Corning (Corning, NY); and type I collagen was obtained from Shengyou Biotechnology Co., Ltd. (Hangzhou, China). Monoclonal type I collagen antibody, fibronectin antibody, and α-smooth muscle actin (α-SMA) antibody were purchased from Abcam (Cambridge, UK). Horseradish peroxidase-conjugated secondary antibody and FITC-labeled secondary antibody was purchased from Beijing Biosynthesis Biotechnology Co., Ltd (Beijing, China). The enhanced chemoluminescence kit (20X LumiGLO® Reagent and 20X Peroxide) was purchased from Cell Signaling Technology, Inc. (Beverly, MA). The protein assay kit (Quick Start, Bradford) was purchased from Bio-Rad (Hercules, CA). The PPARγ agonist, pioglitazone, was purchased from Shandong Zhongke Taidou Chemical Co., Ltd. (Jinan, China). All reagent grade chemicals were from Sigma. Co. (St. Louis, MO) unless otherwise indicated.

### Corneal fibroblast culture

Cultures of rabbit corneal fibroblasts were established by outgrowth from corneal explants, as described previously [3]. Briefly, epithelial and endothelial cells were removed from corneas, the stroma was cut into cubes of approximately 1 mm^3^, placed in Dulbecco’s Modified Eagle’s Medium containing 1 mM NaHCO_3_, and buffered with 25 mM HEPES at pH 7.4. The medium was supplemented with 10% heat-inactivated fetal bovine serum and antibiotics (100 Units/ml penicillin, 50 mg/ml streptomycin-G). Cell cultures between passages 3 and 5 were used for all experiments. The PPARγ agonist, pioglitazone, was prepared as a 1 mM stock in dimethyl sulfoxide (DMSO) and added to cell cultures to the final concentrations indicated. DMSO was added to negative control wells at a final concentration of 0.1%. Cells were treated with pioglitazone at the concentrations of 0.1, 1.0, 3.0, and 10 mM.

### Immunofluorescence staining for α-SMA

To identify the cell phenotype transformation of the keratocytes cultured in serum-containing medium, immunofluorescence staining for α-SMA was performed. Cells at the third passage were grown on coverslips. The medium was removed, and the cell layer was rinsed with PBS. Cells were fixed with 4% paraformaldehyde and permeabilized with triton-100 for 10 min at 20 °C, then were rehydrated with PBS, and then blocked with 5% BSA in PBS for 1 h. Coverslips were sequentially incubated with monoclonal α-SMA antibody and FITC-labeled secondary antibodies, each for 60 min at room temperature. After washing, the nuclei were counterstained with DAPI. Immunofluorescence was visualized using an Olympus microscope and recorded with a high-resolution Olympus digital camera. Negative control was performed using nonimmune serum instead of the primary antibodies.

### Cell viability assessment

The effect of pioglitazone on cell viability was assessed by a 3-[4,5-dimethylthiazol-2-yl]-2,5-diphenyltetrazolium bromide (MTT) assay. Briefly, the corneal fibroblasts were seeded in a 96-well plate at a density of 5,000/well and incubated for 24 h at 37 °C in a humidified atmosphere of 5% CO_2_. Then the cells were treated with pioglitazone at different concentrations (0.1, 1.0, 3.0, and 10 μm), using the culture medium by itself as a control. After a 24 h incubation, the MTT (5 mg/ml in PBS) was added to the culture medium at the rate of 20 μl/well. After another 4 h of incubation at 37 °C in 5% CO_2_, the media were removed and the formazan crystals were solubilized with 100 ml DMSO for 15 min. The optical absorbance was then measured at 570 nm on a microplate reader (Model 680 microplate reader, Bio-Rad). For each concentration, the experiment was performed in triplicate.

### Collagen gel contraction assays

To test the effect of pioglitazone on fibroblast contractility, another function of fibroblasts, fibroblast-mediated gel contraction was measured. Collagen gels were prepared according the instructions from the manufacturer of the type I collagen. Briefly, sterile type I collagen solution, distilled water, Dulbecco’s Modified Eagle’s Medium, and fibroblast suspensions were mixed by pipetting so that the final mixture resulted in 1 mg/ml of collagen and 3×10^5^ fibroblasts/ml gel. A 300 μl portion of the gel solution was then cast into each well of a 24-well culture plate. Collagen lattices were polymerized for 20 min in humidified 5% CO_2_ atmosphere at 37 °C, then incubated with Dulbecco’s Modified Eagle’s Medium containing 10% FBS for 4 h, followed by overnight incubation in serum-free Dulbecco’s Modified Eagle’s Medium. Pioglitazone was added into the culture medium at different concentrations and incubated for 24 h. To initiate collagen contraction, polymerized gels were gently released from the underlying culture dish. To determine the degree of collagen gel contraction, the area of each gel was recorded photographically at day 1, 3, and 5 after release, and was analyzed with Quantity One software (Bio-Rad). Data were expressed as a percentage of the uncontracted gel size.

### Scratch wound closure assays

Confluent cell monolayers in a 6-well plate were wounded by mechanical scraping with a pipette tip. Wound width was assessed at the time of scraping to ensure that all wounds were the same width at the start of the experiments. The cell culture medium was then replaced with fresh medium, with or without pioglitazone treatment, and wound closure was recorded photographically over time, using phase-contrast microscopy.

### Gelatin zymography

Confluent cells grown in 96-well plates were exposed to pioglitazone at different concentrations for 72 h. The culture supernatants were harvested at 72 h and mixed with gel sample buffer. Equal volumes were loaded onto a 10% SDS polyacrylamide gel that contained 1 mg/ml gelatin, and were subjected to electrophoresis. After electrophoresis, the gels were washed in 50 mM Tris buffer that contained 2.5% Triton X-100. The gels were incubated for an additional 40 h in 50 mM Tris buffer (pH 7.6) that contained 5 mM CaCl_2_, after which the gels were stained with Coomassie blue and subsequently destained in methanol/acetic acid/H_2_O. Gels were scanned, and density analysis of the bands was performed using Quantity One software.

### Western blot analysis for collagen I and fibronectin

Confluent cells grown in 6-well plates were exposed to either no pioglitazone (control) or pioglitazone at the concentration of 1, 3 and 10 μm for 72 h. For extracting protein from cell lysates, cells first were washed twice with PBS. They were then lysed in lysis buffer that contained 50 mM Tris-HCl (pH 7.4), 150 mM NaCl, 0.2 g/l sodium azide, 5 g/l sodium deoxycholate, 100 mg/l phenylmethyl sulfonylfluoride, 0.1% SDS, 1% NP-40 and proteinase inhibitor aprotinin (2 mg/l; Amresco Inc., Solon, OH), and leupeptin (2 mg/l; Amresco Inc.) for 20 min. This was followed by centrifugation at 12,000× g for 10 min at 4 °C. Protein concentrations were determined by the Bradford method using a protein assay kit. Protein (50 μg) from cell lysates was mixed with sample buffer, separated on 10% SDS polyacrylamide gels, transferred to nitrocellulose membranes (Pall Life Sciences, East Hills, NY) and then blocked overnight at 4 °C in TBS containing 5% defatted milk powder and 0.1% Tween 20. The membranes were probed with either specific anti-type I collagen and anti-fibronectin primary antibodies, and subsequently washed with TBS-T and incubated with the secondary antibodies conjugated with horseradish peroxidase for 1 h at room temperature. GAPDH immunoblotting was performed as an internal control for equal loading. After extensive washing in TBS-T, the blots were developed for 30 to 60 s with enhanced chemiluminescence reagents. All reagent grade chemicals were from Sigma. Co. (St. Louis, MO) unless otherwise indicated.

### Statistical analysis

Values are means±SD from three to six samples. Comparisons between two groups were analyzed using Student’s t test, and among three groups by ANOVA, followed by a post hoc comparison using the least significant difference test (SPSS 11.5). Values of p<0.05 were considered statistically significant.

## Results

### Transformation of keratocytes to fibroblasts and myofibroblasts

**Figure 1:**
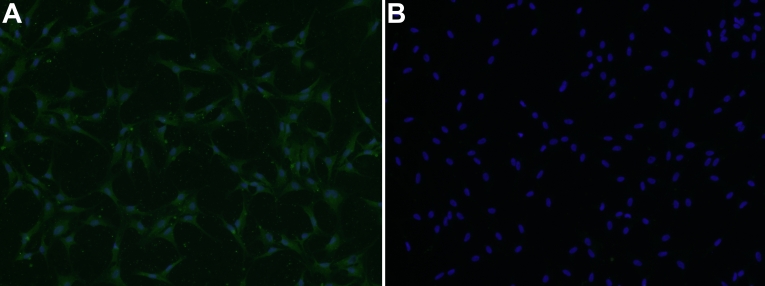
Expression of α-smooth muscle actin in corneal myofibroblasts. The third passage corneal fibroblasts cultured on coverslips were washed with PBS, fixed with 4% paraformaldehyde, blocked in BSA/PBS, and sequentially incubated with monoclonal α-smooth muscle actin and FITC-conjugated secondary antibody. The nuclei were counterstained with 4'-6-diamidino-2-phenylindole(DAPI). Negative controls were performed using nonimmune serum instead of primary antibodies. **A**: Co-localization of α-SMA (green) and nuclei (blue) in myofibroblasts. **B**: Negative control.

The cells, observed with an inverted phase-contrast microscope, exhibited a uniform fibroblast-like shape. Most of the cells were positive for α-SMA staining by immunocytochemistry (Figure 1A). A negative control (normal nonimmune serum was used instead of primary antibody) showed no positive staining (Figure 1B), demonstrating that the cells we used for the experiment were mainly myofibroblasts.

### Pioglitazone inhibits fibroblast motility

**Figure 2:**
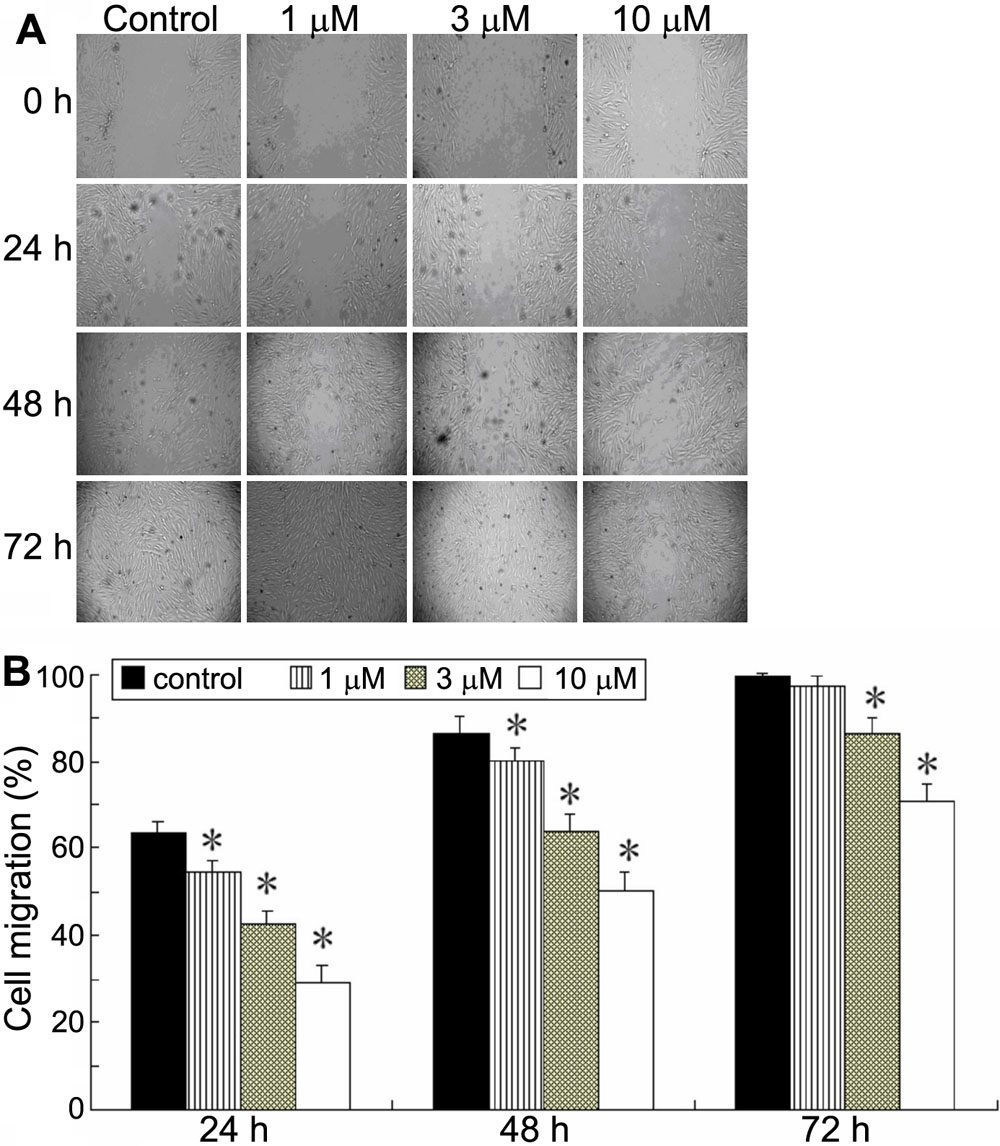
Effect of plioglitazone on corneal fibroblast cell motility. A monolayer of confluent corneal fibroblasts was scraped with a sterile pipette tip after being preincubated with increasing concentrations of pioglitazone (1, 3, and 10 μm) for 24 h. Wound closure was observed by phase-contrast microscopy and photographed at 0, 24, 48, and 72 h. **A**: Photographs of one representative experiment. **B**: Graphic representation of the mean of four experiments. For each sample, the result is expressed as percentage of the wound width at the start of the experiment. The asterisk indicates p<0.05 versus the control group

The effects of pioglitazone on corneal fibroblast migration were assessed by scrape-wound assay. As shown in Figure 2A, photomicrographs taken 24, 48, and 72 h after wounding showed delayed wound closure by corneal fibroblast cultures treated with pioglitazone at concentrations ranging from 1 to 10 μm compared with untreated control cultures. Quantitation of the wound closure over time revealed a significant inhibitory effect of pioglitazone on corneal fibroblast motility (Figure 2B) at even the lowest concentration of pioglitazone (1 μm).

### Pioglitazone reduces fibroblasts’ capacity to contract free-floating collagen lattices

**Figure 3:**
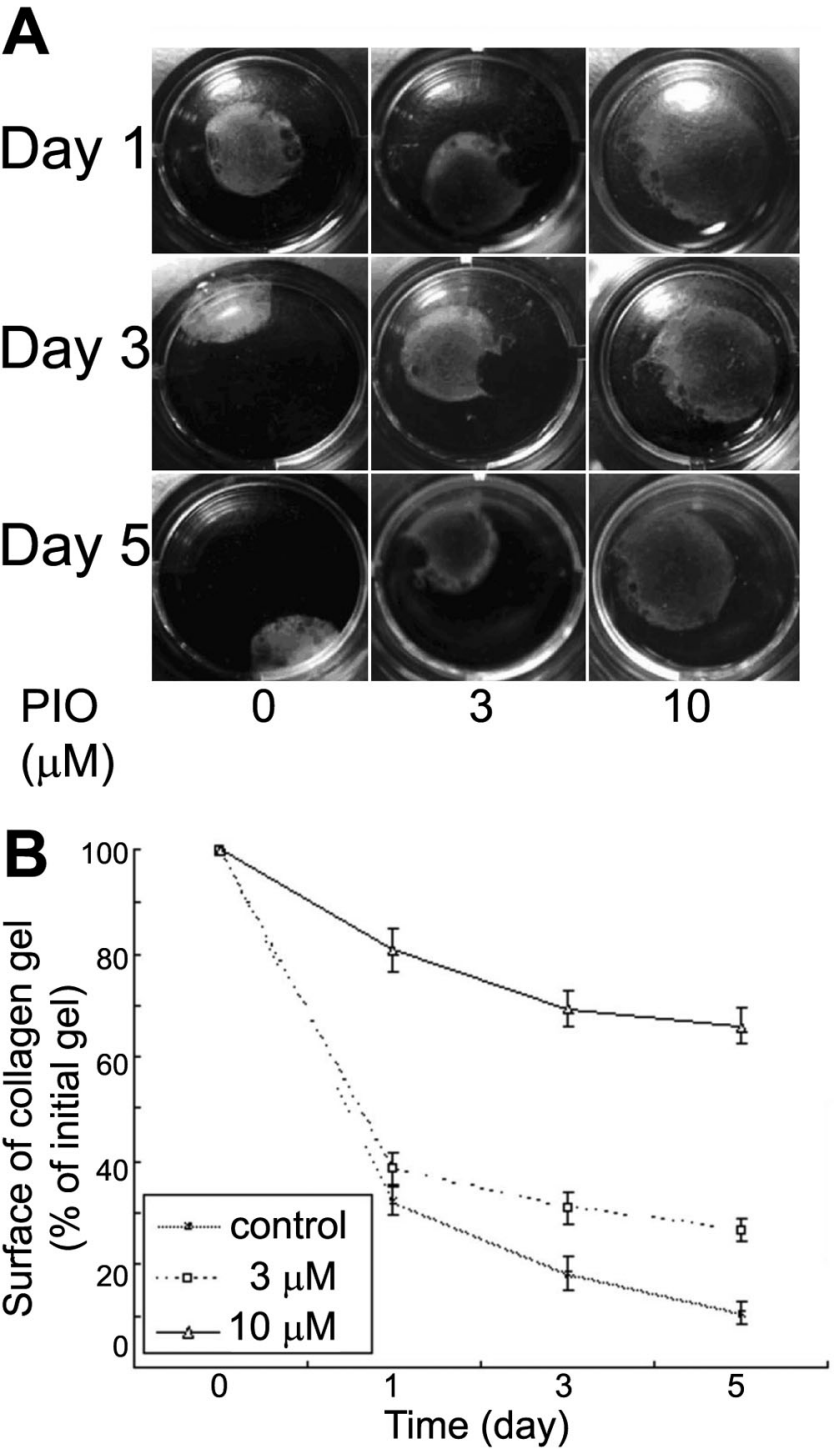
Pioglitazone inhibited the ability of corneal fibroblasts to contract free-floating collagen lattices. Corneal fibroblasts were seeded in collagen gel lattice in the absence or presence of pioglitazone at the concentrations of 3 and 10 μm. Cell contractility was assessed by measuring the reduction in the surface area of the floating collagen gel discs for the times shown (1–5 d). **A**: Photographs of one representative experiment. **B**: Graphic representation of the mean±SD of three experiments.

To determine the effect of pioglitazone on ECM contraction induced by fibroblasts, corneal fibroblasts were seeded in free-floating collagen gels and incubated in the presence or absence of piogliatazone. The kinetics of collagen lattice contraction were then recorded over a 5 d period. Results demonstrated that pioglitazone had a potent dose-dependent inhibitory effect on collagen lattice contraction by corneal fibroblasts, especially at the concentration of 10 μm, as shown in Figure 3. At day 5, pioglitazone treatment at concentrations of 3 and 10 μm resulted in significantly greater lattice diameters, relative to the control.

### The effect of pioglitazone on MMP-2 and MMP-9 secretion

**Figure 4:**
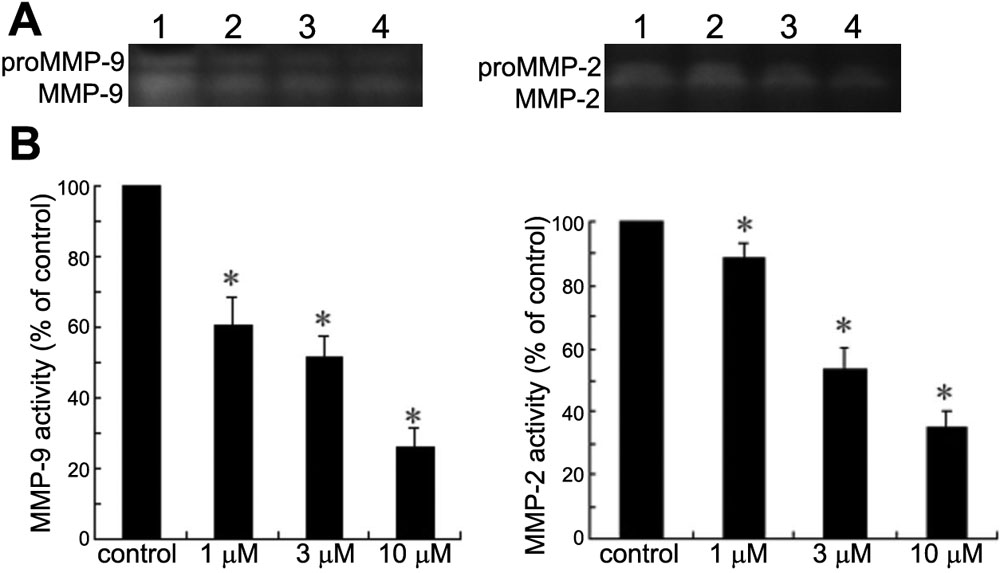
Detection of MMP-2 and MMP-9 by gelatin zymography. Treatment with pioglitazone at concentrations of 1, 3, and 10 μm resulted in significant decreases in MMP-2 and MMP-9 secretion. **A**: A representative zymogram is shown. Lane 1, control; lane 2, 1 μm; lane 3, 3 μm; lane 4, 10 μm. **B**: The densitometric values of the MMP-9 and MMP-2 bands are expressed as percentages of the control. Results are the mean±SD of three independent experiments (the asterisk indicates p<0.05 versus control), indicating a dose-dependent inhibition of pioglitazone on MMP-9 and MMP-2 secretion.

After exposure to pioglitazone at different concentrations, there was a decrease in the cellular secretion of both MMP-9 and MMP-2, detected by gelatin zymography, as shown in Figure 4. MMP-9 activity was reduced by 41%, 48%, and 74%, and MMP-2 was reduced by 11%, 47%, and 65%, compared with the control after treatment with pioglitazone at the concentrations of 1, 3, and 10 μm, respectively.

### Effect of pioglitazone on type I collagen and fibronectin expression

**Figure 5:**
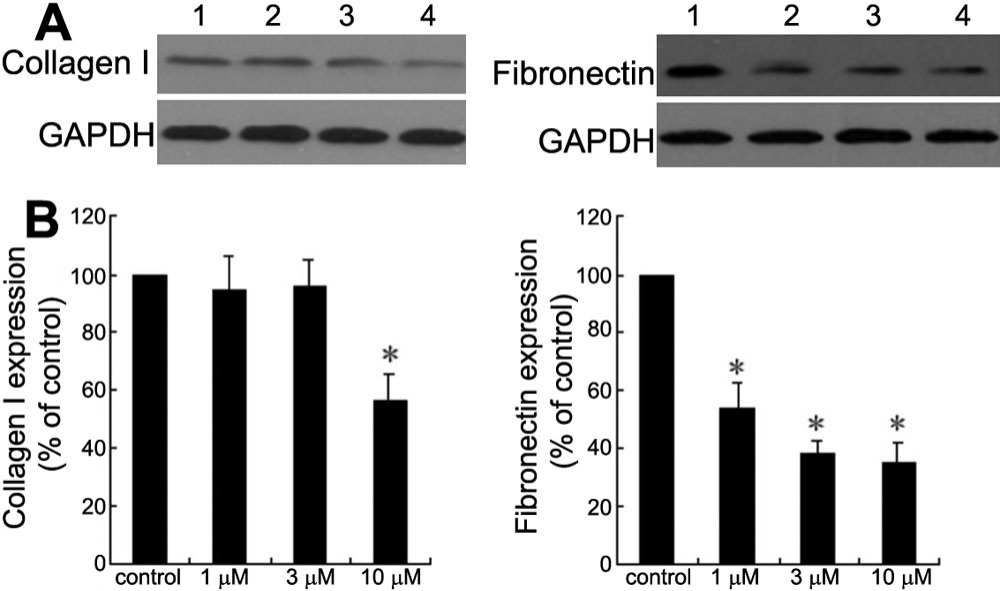
Effect of pioglitazone on collagen I and fibronectin expression. The expression of collagen I and fibronectin by corneal fibroblasts was analyzed by western blotting after treatment with or without pioglitazone (1, 3, and, 10 μm). **A**: Representative immunoblot for collagen I and fibronectin. Lane 1, control; lane 2, 1 μm; lane 3, 3 μm; lane 4, 10 μm. **B**: Quantitative analysis of collagen I and fibronectin protein expression by densitometry from cells described in **A**. Values are the means±SD from three separate experiments. The asterisk indicates p<0.05 compared with control.

To determine the effect of PPARγ agonists on corneal fibroblast production of collagen I and fibronectin, cells were treated with pioglitazone, and western blotting was performed to detect the expression of collagen I and fibronectin. As shown in Figure 5, the results indicated that fibronectin protein expression was reduced by pioglitazone in a dose-dependent manner, while collagen I synthesis was only significantly reduced at a relatively high concentration of pioglitazone (10 μm).

### Pioglitazone has no evident effect on the viability of corneal fibroblasts

**Figure 6:**
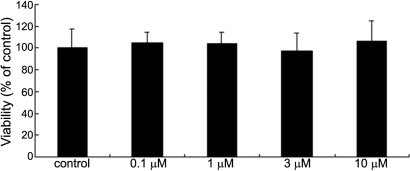
Cell viability is not influenced by treatment with pioglitazone. After treatment with medium alone, or with pioglitazone at 0.1, 1, 3, and 10 μm, respectively, for 72 h, the viability of corneal fibroblasts was measured by MTT assay. Results are shown as percentages of the control (untreated cells). There was no significant difference between any treatment (ANOVA) p>0.05 versus control.

To exclude the possibility that the anti-fibrotic effect of pioglitazone was mediated by cellular toxicity, MTT assay was used to examine the viability of corneal fibroblasts after treatment with pioglitazone at different concentrations. Viable cells actively cleave the MTT reagent and form a colored precipitate, the appearance of which is proportionate to the number of viable cells. Results are expressed as a percentage of the negative control, the cells with only media. As shown in the Figure 6, there was no significant difference in cell viability between the pioglitazone treated group and the control. This suggests pioglitazone might exert its antifibrotic effect in a non-cytotoxic way.

## Discussion

The cornea is composed of three tissue layers: the outer stratified squamous epithelium, the inner endothelium, and the intermediate stroma. The stroma makes up 90% of the corneal thickness. It is comprised of collagen fibers that are arranged in bundles, and of sparsely distributed stromal cells that are referred to as keratocytes. This particular structure is the basis for the maintenance of corneal transparency. However, various kinds of injuries, including inflammatory reactions and fibrotic responses, will destroy the homeostasis of the cornea and induce the repair process. It must be emphasized that although corneal fibrosis is very important for the healing of injured corneas, over-reactive fibrosis usually gives rise to many undesirable effects. Previous research has shown that corneal fibrosis can lead to loss of transparency to different extents, due to the lack of precise organization, and to molecular changes in the repair tissue and cells. It can also result in changed corneal shape, due to repair tissue contraction.

So far, there is no effective and safe strategy for the prevention or inhibition of corneal scar formation in clinical practice. Corticosteroid is now widely used for its anti-inflammatory effect, and it does have a significant inhibitory effect on fibrotic reactions. However, it is generally recognized that it can be hardly used in the acute phase of infection because of its suppression of the immune system. It can also give rise to the risk of glaucoma and cataracts. Therefore, we should explore novel approaches to regulating fibrotic reactions during the corneal repair process.

Peroxisome proliferator-activated receptors (PPARs) comprise a family of ligand-activated transcription factors belonging to the nuclear hormone receptor family. They are related to retinoid, glucocorticoid, and thyroid hormone receptors [15]. There are three subtypes, PPARα, PPARβ, and PPARγ, with a wide range of effects on metabolism, cellular proliferation, and immune responses [16,17]. PPARs were first identified in their role in lipid and glucose regulation, and until recently, many research studies have demonstrated that PPARs have anti-fibrotic, anti-inflammatory, and other important functions. Fibrosis-related diseases are involved in almost all connective tissues, and share common pathological characteristics. Fibrosis in many tissues, such as the kidney, liver, lung, heart, and pancreas, has been demonstrated by experiments to be inhibited by the activation of PPARγ. Moreover, fibrosis in ocular tissues, including corneal fibrosis and conjunctival fibrosis, has also been demonstrated to be suppressed by PPARγ activation in animal models [18,19]. However, the cellular and molecular mechanism of the anti-fibrotic action of PPARγ activation in corneal tissue is not yet well understood.

To investigate the mechanism by which PPARγ agonist exerts an anti-fibrotic effect on corneal fibrosis, we took corneal keratocytes cultured in a serum-containing condition as an in vitro model of corneal wound healing. The phenotypic transition from keratocyte to repair cell in corneal wounds is similar to the change that occurs when keratocytes are isolated from the normal stroma, placed in a cell culture, and exposed to serum [20,21]. The transition is characterized by a change in cell shape, exhibiting many morphological characteristics of fibroblasts, including a fusiform shape, multiple nucleoli, and a lack of cytoplasmic granules [22].

In our study, pioglitazone was shown to inhibit the corneal fibroblast migration, and to reduce their capacity to contract free-floating collagen lattices, which is thought to be related to the expression of α-SMA. Moreover, the secretion of MMP-2 and MMP-9 in the supernatant of cell cultures decreased after the subministration of pioglitazone. The MMPs are believed to play an important role in corneal remodeling after wounding, through its critical effect on the balance between matrix degradation and production. Collagen I and fibronectin are the major components of corneal extracellular matrices, the expression of which clearly increases during the process of wound healing. Our results show pioglitazone significantly inhibited the expression of fibronectin, but only had an inhibitory effect on collage I expression at a relatively high concentration. We can therefore deduce that PPARγ activation might influence the extracellular matrix turnover in two different aspects: those involving production and degradation.

Cell viability assay in this study suggested that the antifibrotic effect of pioglitazone might operate in a non-cytotoxic manner. The signaling pathway in the antifibrotic action of PPARγ activation is still not entirely understood. Li et al. [23] proved that hepatocyte growth factor (HGF) is a downstream effector that mediates the antifibrotic action of peroxisome proliferator–activated receptor-γ agonists in their study on renal fibrosis lesions. In addition, several research studies have demonstrated that crosstalk between PPARγ and TGF-β1, Smad2/3, and JNK signaling pathways is critical for the suppression of Ang II-induced production of PAI-1 and ECM [24,25].

It must be pointed out that there exist several limitations to this study. Our study has demonstrated the anti-fibrotic effect of the PPARγ agonist on cultured corneal fibroblasts in vitro, which is similar to the active phenotype in the corneal repair process. However, what effect might it have on the transformation of keratocyte to fibroblast or myofibroblast? This is the topic of research that we are now investigating; moreover, the in vivo effect of PPARγ activation remains to be further investigated.

Overall, our results demonstrate that pioglitazone, a PPARγ activator, has an evident anti-fibrotic effect on corneal fibroblasts, cultured in vitro, by such mechanisms as suppression of cell motility, contraction, and expression of the extracellular matrix, as well as the secretion of MMPs. These data are exciting, and suggest that the PPARγ pathway is likely a very important future target for corneal fibrosis therapy, and that it might be used to attenuate scar formation during the corneal repair process.
